# Demethylated miR-216a Regulates High Mobility Group Box 3 Promoting Growth of Esophageal Cancer Cells Through Wnt/β-Catenin Pathway

**DOI:** 10.3389/fonc.2021.622073

**Published:** 2021-03-23

**Authors:** Cheng-Xi Sun, Feng Zhu, Lei Qi

**Affiliations:** ^1^ Department of Clinical Laboratory, Cheeloo College of Medicine, Shandong University, Jinan, China; ^2^ Department of Thoracic Surgery, Shandong Provincial Chest Hospital, Jinan, China; ^3^ Department of Thoracic Surgery, Cheeloo College of Medicine, Shandong University, Jinan, China

**Keywords:** esophageal cancer, miR-216a, DNA methylation, HMGB3, Wnt/β-catenin pathway

## Abstract

**Background:**

Esophageal cancer (EC) is the eighth most common cause of cancer-associated mortality in humans. Recent studies have revealed the important roles of microRNAs (miRs) in mediating tumor initiation and progression. miR-216a has been found to be involved in the progression of EC, but the underlying mechanisms remain largely unknown. The aim of this study is to explore the mechanism of miR-216a and the downstream molecules in esophageal cancer.

**Materials and Methods:**

The degree of methylation of miR-216a promoter in EC tissues and cell lines was determined with methylation specific polymerase chain reaction (MSP). The levels of miR-216a and HMGB3 in EC cells were quantified by quantitative PCR (qPCR) and Western blot (WB). EC cell lines were treated with DNA methylation inhibitor 5-aza-2’-deoxycytidine (5-AZ), miR-216a mimics, and HMGB3 siRNA to explore the effects of miR-216a and HMGB3 on the proliferation, migration, invasion, and apoptosis of cells. Dual-luciferase reporter assay was employed to verify the binding of miR-216a to the 3’UTR of HMGB2 mRNA.

**Results:**

The promoter of MiR-216a was hypermethylated and the expression of miR-216a was down-regulated in EC, while HMGB3 was up-regulated. Dual luciferase reporter assay confirmed the binding of miR-216a to the 3’UTR of HMGB3 mRNA. Demethylated miR-216a and miR-216a mimics elevated miR-216a expression and down-regulated HMGB3, as well as inhibited cell proliferation, migration, and invasion. Inhibiting the expression of HMGB3 played an important role in inducing apoptosis, suppressing cell expansion, and down-regulating the activity of Wnt/β-catenin pathway.

**Conclusions:**

Hypermethylation in the promoter of miR-216a upregulated HMGB3 and the Wnt/β-catenin pathway, resulting in enhanced EC progression.

## Introduction

Esophageal cancer (EC), a common cancer with high metastasis (1), is mainly classified into esophageal adenocarcinoma (EAC) and esophageal squamous cell carcinoma (ESCC). Although ESCC is more prevalent than EAC at present (2), the incidence of EAC is rising (3). Due to the poor prognosis and low survival of EC (4, 5), improving the treatment efficacy has become the clinical focus. Esophageal carcinogenesis is associated with microRNA (miRNA) regulation (6–9), so understanding the expression and function of miRNAs in EC is helpful to promote the early diagnosis and treatment of EC.

MiR-216a is a 110 bp miRNA located on human chromosome 2. It is involved in a variety of cellular processes and emergence of diseases through pairing and binding with different target genes (10–13). Moreover, it has been reported to be down-regulated in EC, which improves cancer cell viability and promotes the progression of EC (14, 15). High mobility group box 3 (HMGB3), one of the members of the high-mobility group family, is an important regulator of stem cell self-renewal and differentiation (16). Its abnormal expression or dysfunction leads to the imbalance of cellular homeostasis. Besides, HMGB3 plays a role in regulating cell proliferation and apoptosis, and its abnormal expression may result in malignant phenotype of breast cancer (17–22). Unlike the down-regulation of miR-216a, HMGB3 is highly active in EC (23). The wnt/β-catenin pathway is one of major pathways contributing to EC progress, which mainly includes β-catenin, c-myc, and MMP7. Wnt/β-catenin level could help to estimate the progress of EC in molecular biology.

Here, we found CpG islands in miR-216a promoter were methylated through Methprimer prediction and methylation specific PCR. Hypermethylation of miR-216a resulted in up-regulation of HMGB3 through direct targeting of the 3’UTR, and then activated the Wnt/β-catenin pathway and promoted malignant proliferation and metastasis of EC.

## Materials and Methods

### Patients With EC

Cancer tissues from 82 patients with EC in Qilu Hospital of Shandong University were sampled. All patients were diagnosed with EC based on clinical features or biopsy and were free of mental diseases, other tumors, or history of EC treatment. The patients were fully informed, and were willing to cooperate with the therapist. The Ethics Committee of Qilu Hospital of Shandong University approved the study and this study is in line with the Declaration of Helsinki. Tissue sections were stored in −80° before testing. In addition, 68 normal adjacent tissues were chosen to be control group.

### Cell Culture and Transfection

Human normal esophageal epithelial cells (HET-1A) and EC cell lines (TE-1, TE-9, KYSE30, EC9706) were purchased from ATCC. Culture medium for EC cell lines: Dulbecco’s Modified Eagle Medium (DMEM, Hyclone) plus 10% fetal bovine serum (FBS, Gibco) and 1% penicillin/streptomycin solution (100X, Solarbio). The cells were incubated in a humidified incubator at 37° in with 5% CO2.

Before transfection, the medium was replaced with FBS-free medium. During transfection, 1 × 10^5^ cells were inoculated into each well of six-well plates. MiR-216a mimics (miR mimics), miR-216a inhibitor (miR inhibitor), NC mimics, NC inhibitor, HMGB3 siRNA, NC siRNA vectors were purchased from Sangon Bioengineering (Shanghai, China). The cell lines were transfected using the Lipofectamine 2000 kit (Invitrogen, USA).

### Methyl Thiazolyl Tetrazolium (MTT) Assay

Ninety-six-well plates were used to for cell inoculation, with the cell density at 5 × 10^3^/100 μl per well. The plates were incubated for 24, 48, 72, and 96 h respectively, then added with 5 mg/ml MTT solution (dissolved in dimethyl sulfoxide, Solarbio) at 10 μl/well, and cultured for another 1 h. Afterwards, the medium was removed, and the optical density (OD) value at 570 nm was measured by a microplate reader.

### Transwell

The cells (2 × 10^4^/well) were inoculated into the apical chamber (200 μl of DMEM containing 1% FBS), and DMEM (containing 10% FBS, with a total volume of 500 μl) was added into the basolateral chamber. The chamber was cultured at 37° with 5% CO2 for 24 h, then the liquid in the apical chamber was removed and the cells were wiped off. Cells attached to the opposite side of the membrane were fixed in 4% paraformaldehyde for 20 min, then stained for 15 min with crystal violet. Afterwards, the Transwell chamber was washed with phosphate buffer saline (PBS) buffer. The migrated cells were counted in three random visual fields under a light microscope (100× magnification), and the average value was calculated. The experiment was repeated three times. As for the invasion assay, the chamber was covered with 0.5 mg/ml Matrigel matrix (Corning #356235) in serum free medium, the number of cells was increased to 5 × 10^4^ per well, and the rest steps were the same as the migration assay described above. For the control group of cell migration or invasion, the basolateral chamber was added with DMEM containing 1% FBS.

### Methylation-Specific PCR (MSP) and qPCR

Cancer tissue or adjacent normal tissues were prepared into cell suspension, and 1 ml of trizol was added to the suspension to extract total RNAs. The OD values of total RNAs at 260–280 nm was measured by an ultraviolet spectrophotometer, and those with the value of OD260/OD280 >1.8 were enrolled for subsequent qPCR. FastQuant RT Super Mix (KR108) kit (Tiangen Biotech, Beijing, CHN) was applied for reverse transcription.

Methprimer predicted miR-216a CpG loci. The reverse transcription products were treated with sodium bisulfite using an EZ DNA Methylation-Gold Kit (ZYMO RESSEARCH, USA). The MSP kit was purchased from Tiangen Biotech (Beijing, CHN). The miR-216 MSP primer was designed and synthesized by Sangon Bioengineering (Shanghai, CHN). MiR-216 MSP primers, forward (F): 5’-GGA ATA TGG TTT TAT TTT TAT GGG C-3’, reverse (R): 5’-ATA AAA TTT TAT AAT AAT TTC-3’; MiR-216a UMSP primers, F: 5’-GAT AGA GGT GCT GGT TG-3’, R: 5’-CCT AAT CTA TTC CCT A-3’.

FastFire qPCR PreMix (SYBR Green Kit, Tiangen Biotech, Beijing, CHN) and ABI PRISM 7000 (Applied Biosystems, USA) performed fluorescence quantification on miR-216a and HMGB3 mRNA. Primers were designed and synthesized by Sangon Bioengineering (Shanghai, CHN). MiR-216a primer, F: 5’-TGT CGC AAA TCT CTG CAG G-3’, R: 5’-CAG AGC AGG GTC CGA GGT A-3’; HMGB3 primer, F: 5’-GAC CAG CTA AGG GAG GCA A-3’, R: 5’-ACA GGA AGA ATC CAG ACG GT-3’. Results were analyzed by ABI PRISM 7000. U6 and GAPDH were served as internal reference genes, and the data were normalized by 2^–ΔΔCt^.

### Western Blot

After harvest, cells were lysed in 20 mmol/L Tris-HCl (pH 7.5) plus protease inhibitor (Solarbio). The extract was centrifuged at 4° for 20 min at 16,000 *g*. Protein concentration in the supernatant was determined by bicinchoninic acid (BCA) method. The samples were separated by SDS-PAGE. Afterwards, the separated protein was then transferred onto nitrocellulose (NC) membrane. Non-specific binding sites were blocked with 5% skim milk. The membrane was probed with primary and secondary antibodies and then visualized with enhanced chemiluminescence (ECL) reagent. β-actin was used as the internal reference, and the relative expression level of the target protein was presented as gray value of test band/gray value of the β-actin band. Primary antibodies of HMGB3 (ab18256), Caspase 3 (ab32351), Caspase 9 (ab138412), E-cadherin (ab231303), B-cell lymphoma-2 (Bcl-2) (ab182858), BCL2-Associated X (Bax) (ab32503), N-cadherin (ab76011), β-catenin (ab32572), c-myc (ab32072), Matrix metalloproteinase 7 (MMP7) (ab207299), β-actin (ab8226), and secondary antibody (HRP conjugate) were all purchased from Abcam.

### Dual-Luciferase Reporter Gene Assay

Targetscan7.2 was applied to predict the target site of miR-216a in the 3’UTR of HMGB3 mRNA. Cells were inoculated into 96-well plates. GLO-HMGB3-wild type (wt) and GLO-HMGB3-mutant (mut) vectors were constructed and co-transfected with miR-216a mimics and NC mimics respectively. After 48 h of transfection, luciferase activity was detected in a dual-luciferase reporter assay system (Promega).

### Statistics

The data were statistically analyzed with SPSS20.0 (Asia Analytics Formerly SPSS China), and visualized with GraphPad Prism 6.0. Each experiment was repeated for three times, and the results were expressed by Mean ± standard deviation (SD). Independent samples t test was used to compare the statistical differences between EC tissues and normal adjacent tissues, NC siRNA group, and HMGB3 siRNA group. One-way analysis of variance (ANOVA) was adopted for multi-group comparison, and Fisher’s least significant difference-t test for *post-hoc* pairwise comparison. All data were analyzed with two-tailed test. Ninety-five percent was taken as its confidence interval. P < 0.05 was considered statistically significance.

## Results

### Hypermethylation of miR-216a Down-Regulates Its Expression in EC

**Figure 1:**
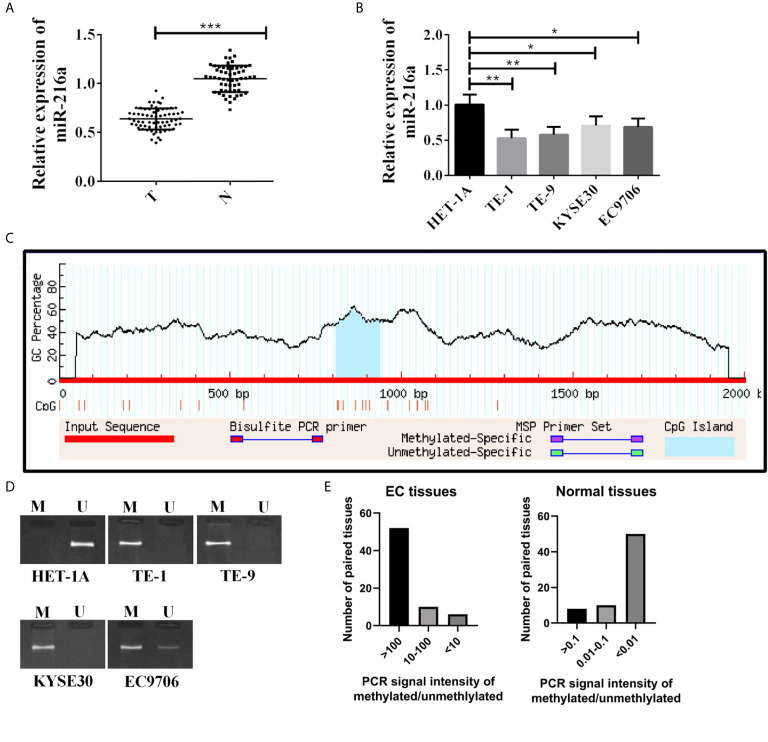
MiR-216a is down-regulated and methylated in the promoter region in esophageal cancer. **(A)** MiR-216a is down-regulated in esophageal cancer tissues. Sixty-eight paired EC tissues and normal adjacent tissues were analyzed by qRT-PCR. ***P < 0.001. T, tumor; N, normal adjacent tissue. **(B)** MiR-216a is down-regulated in EC cell lines. N = 3, *P < 0.05, **P < 0.01, ***P < 0.001. **(C)** Prediction of CpG island in the promoter region of miR-216a. The promoter of miR-216a is about 2 kb in length. CpG island was predicted near the center of the promoter (from 809 to 934 bp) by Methprimer. **(D)** MiR-216a promoter is methylated in EC cell lines. M represented the PCR amplification product using methylation specific primers. U indicated the amplification product using conventional primers. **(E)** MiR-216a promoter is methylated in EC tissues from patients. MSP was performed in 68 patient tissues. The intensity of methylation band was divided by the unmethylation band to calculate the methylation/unmethylation values of 68 samples and used for plotting.

Sixty-eight EC tissues and 68 paired normal adjacent tissues were used in this study. qPCR quantified miR-216a mRNA expression level, and down-regulation of miR-216a was found in EC tissues (**Figure 1A**). Then, miR-216a levels in normal cell lines and EC cell lines were analyzed. Similarly, miR-216a was down-regulated in EC cell lines (**Figure 1B**). The methylation status of miR-216a promoter was predicted by Methprimer to explore the underlying mechanism of down-regulation of miR-216a in EC. The results showed that there was CpG island at 809-934 bp of miR-216a promoter (**Figure 1C**), which could be hypermethylated in EC tissue and cell line. After that, MSP for the determination of methylation degree of miR-216a promoter illustrated that methylation bands appeared in EC cell samples (**Figure 1D**). Likewise, we analyzed the 68 paired EC tissues and normal adjacent tissues using MSP and found that strong methylation bands appeared in more than 75% of the cancer tissues (**Figure 1E**). The above findings suggested that hypermethylation of miR-216a was associated to its down-regulation in EC.

### MiR-216a Regulated the Expression of HMGB3 Through Direct Targeting

**Figure 2:**
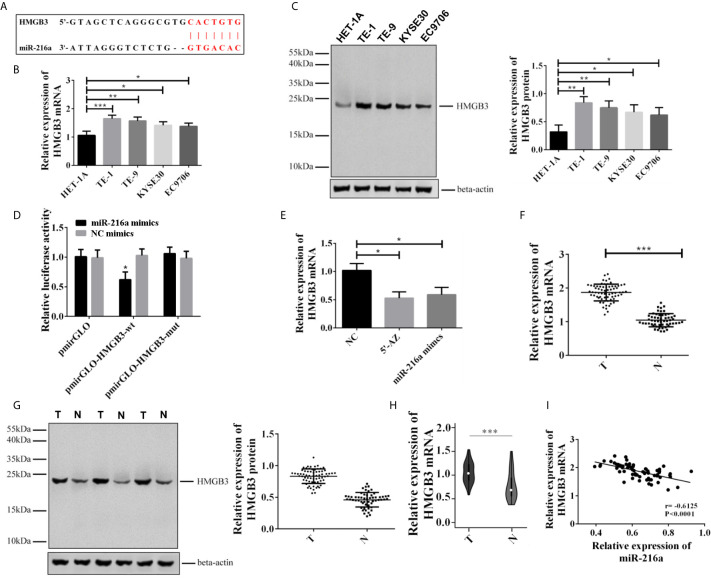
miR-216a targeted to the 3’UTR of HMGB3 and down-regulated its expression in EC tissues and cell lines. **(A)** Predicted binding loci of miR-216a in the 3’UTR of HMGB3 mRNA. The red color indicates base pairs; **(B, C)**: HMGB3 was up-regulated in EC cell lines as detected by qRT-PCR and western blot. Representative western blot data were shown. N = 3–4. *P < 0.05, **P < 0.01, ***P < 0.001. **(D)** Luciferase activity decreased when TE-1 cells were co-transfected with miR-216a mimics and the plasmid construct containing the wildtype 3’UTR of HMGB3 (HMGB3-wt), compared with NC mimics group. In contrast, the plasmid construct containing the mutated 3’UTR at the miR-216a binding sites (HMGB3-mut) showed similar levels of luciferase activity. The luciferase activities of the empty plasmid control (pmirGLO) co-transfected with either miR-216a mimics or NC were also unchanged. N = 3, *P < 0.05. **(E)** Inhibition of DNA methylation by 5-AZ or up-regulating miR-216a by miR-216a mimics inhibits HMGB3 mRNA expression (N = 3, *P < 0.05). **(F, G)** HMGB3 was up-regulated in esophageal cancer tissues as detected by qRT-PCR and western blot. Sixty-eight paired EC tissues and normal adjacent tissues were analyzed by qRT-PCR and western blot. The representative western blot data were three pairs of tissue samples. ***P < 0.001. T, tumor; N, normal adjacent tissue. **(H)** HMGB3 was up-regulated in TCGA database. We retrieved 416 patients with EC from the TCGA database who had been diagnosed with EC, and the expression of HMGB3 was analyzed (***P < 0.001). **(I)** Pearson analysis demonstrates that miR-216a is negatively correlated with HMGB3 mRNA.

Since miR-216a was downregulated in EC tissues and cell lines, we sought to identify the downstream target of miR-216a in EC cell lines. Through Targetscan 7.2 prediction, the binding loci of miR-216a were found at 3’ untranslated region (3’UTR) of HMGB3 mRNA (**Figure 2A**). Next, we verified the binding of miR-216a to HMGB3 by dual luciferase reporter assay. miR-216a had the lowest expression in TE-1 cell line (**Figures 2B, C**), thus we selected TE-1 cells for the following study. We found the luciferase intensity decreased when TE-1 cells were co-transfected with miR-216a mimics and HMGB3-wt, but not by HMGB3-mutant with mutated binding sites (**Figure 2D**). Demethylating miR-216a promoter or up-regulating miR-216a inhibited HMGB3 mRNA in EC cell lines (**Figure 2E**). We also checked the mRNA and protein levels of HMGB3 in paired EC tissues and adjacent normal tissues from 68 patients and found that HMGB3 was up-regulated (**Figures 2F, G**). Similarly, the expression of HMGB3 was analyzed in EC patients in TCGA database, and we found that HMGB3 was up-regulated in EC tissues (**Figure 2H**). Pearson analysis demonstrated that miR-216a was negatively correlated with HMGB3 mRNA (**Figure 2I**). Overall, miR-216a binds to the 3’UTR and inhibit HMGB3 expression.

### Up-Regulation of miR-216a Inhibited Invasion, Migration, and Promoted Apoptosis of TE-1 Cells

**Figure 3:**
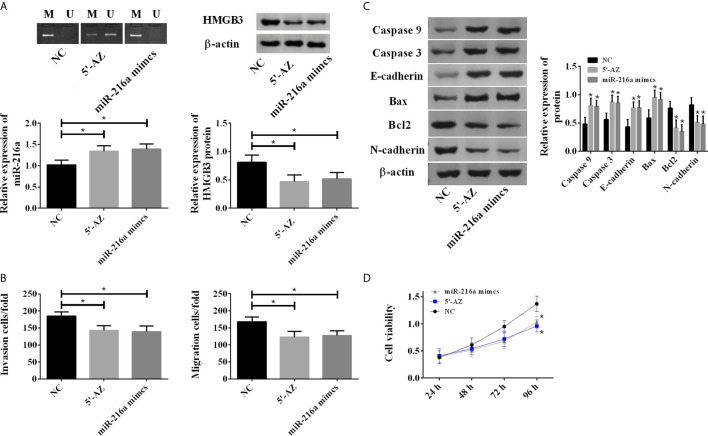
5-AZ promoted the expression of miR-216a and inhibited TE-1 cell survival through down-regulation of HMGB3. **(A)** 5-AZ significantly reduces the methylation of miR-216a and up-regulates the expression, miR-216a mimics exert no effect on the methylation of miR-216a but significantly up-regulate its expression, and both of them down-regulate HMGB3 (N = 3, *P < 0.05). **(B)** Demethylated miR-216a and miR-216a mimics led to reduced cell migration and invasion (N = 3, *P < 0.05). **(C)** Demethylated miR-216a and miR-216a mimics increase Caspase 3, Caspase 9, E-cadherin, Bax and decrease Bcl2 and N-cadherin, compared with NC group. Representative western blot images were shown here, and the uncropped raw data were shown in **Supplementary Figure 1**. The quantification data were Mean ± SD of three independent experiments (N = 3. *P < 0.05). **(D)** Demethylated miR-216a and miR-216a mimics inhibit cell viability, compared with NC group (N = 3, *P < 0.05).

To test whether the hypermethylation of miR-216a promoter reduced its expression, the TE-1 cells were treated with DNA methylation inhibitor (5-aza-2’-deoxycytidine, 5-AZ) and transfection of miR-216a mimics, respectively. 5’-AZ significantly reduced the methylation of miR-216a and up-regulated its expression, while miR-216a mimics exerted no effect on the methylation of miR-216a promoter but significantly up-regulated its expression, and both of them down-regulated HMGB3 (**Figure 3A**). Moreover, we found up-regulation of miR-216a inhibited TE-1 cell migration and invasion (**Figure 3B**), and increased the protein levels of Caspase 3, Caspase 9, E-cadherin, Bax, as well as decreased Bcl2 and N-cadherin in TE-1 cells (**Figure 3C** and **Supplementary Figure 1**). The cell viability was decreased when the expression of miR-216a was up-regulated (**Figure 3D**). Overall, these data suggested that up-regulation of miR-216a inhibited the invasion and migration of TE-1 cells and promoted the apoptosis.

### Effects of miR-216a Were Mediated Through the HMGB3 and Wnt/β-catenin Pathway

**Figure 4:**
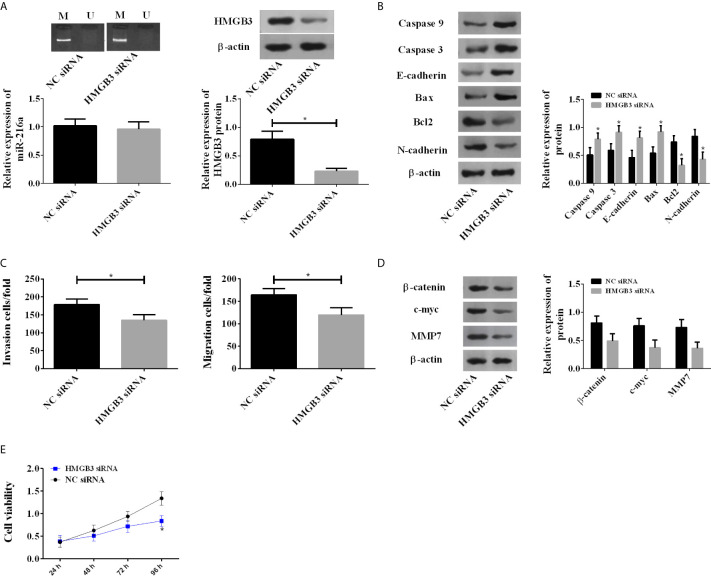
Down-regulation of HMGB3 by siRNA enhanced the expression of Wnt/β-catenin pathway proteins. **(A)** Down-regulation of HMGB3 shows no influences on the methylation degree and expression of miR-216a, but causes a significant decrease in HMGB3 protein. miR-216a levels were measured by qRT-PCR. HMGB3 proteins were detected by western blot, and the representative images were shown. The unprocessed raw data were presented in **Supplementary Figure 2**. (N = 3, *P < 0.05). **(B)** Down-regulation of HMGB3 causes increase of Caspase 3, Caspase 9, E-cadherin, Bax and decrease of Bcl2, N-cadherin, compared with NC siRNA group (N = 3, *P < 0.05). See **Supplementary Figure 3** for the raw data of western blot. **(C)** Down-regulation of HMGB3 inhibits cell migration and invasion (N = 3, *P < 0.05). **(D)** Down-regulation of HMGB3 suppresses Wnt/β-catenin pathway proteins β-catenin, c-myc, and MMP7, compared with NC siRNA group (N = 3, *P < 0.05). See **Supplementary Figure 4** for the raw data of western blot. **(E)** Down-regulation of HMGB3 weakens cell viability, compared with NC siRNA group (N = 3, *P < 0.05).

Due to the abnormal up-regulation of HMGB3, we investigated its roles in EC by using HMGB3 siRNA. Knockdown of HMGB3 showed no influences on the methylation degree and expression of miR-216a, but caused a significant decrease in HMGB3 protein (**Figure 4A**). Knockdown of HMGB3 increased the protein levels of Caspase 3, Caspase 9, E-cadherin, Bax, and decreased Bcl2 and N-cadherin (**Figure 4B**), suggesting enhanced apoptosis. TE-1 cell migration and invasion were also inhibited by HMGB3 knockdown (**Figure 4C**). We analyzed Wnt/β-catenin pathway proteins, including β-catenin, c-myc, and MMP7 by western blot, and found they were decreased (**Figure 4D**). The above results indicated that the down-regulation of HMGB3 inhibited TE-1 cell survival *via* Wnt/β-catenin pathway (**Figure 4E**).

### Rescue Experiment

**Figure 5:**
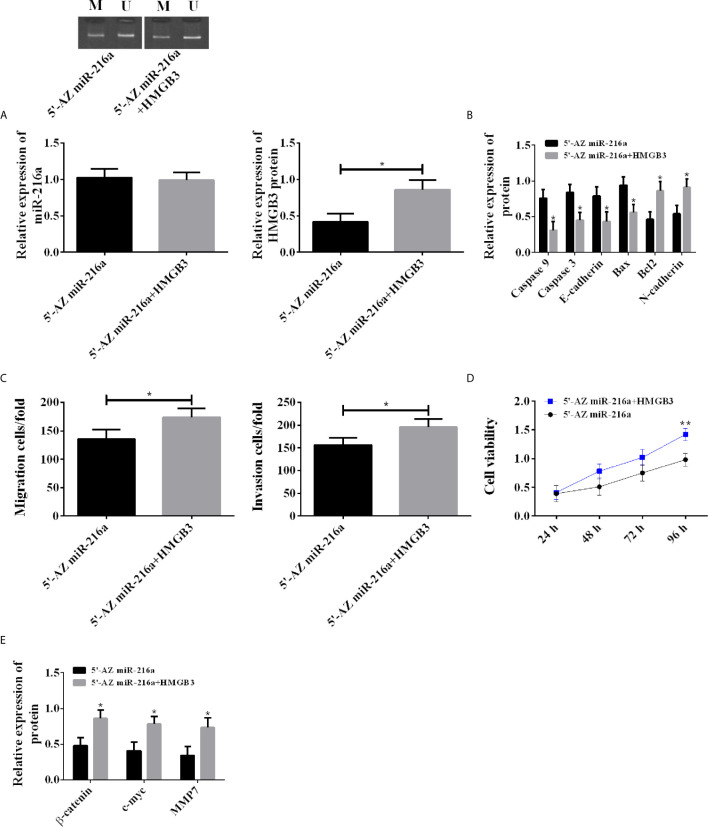
Rescue experiment in TE-1 cell line. **(A)** Up-regulating HMGB3 has no effect on miR-216a expression, but counteracts the down-regulation of HMGB3 induced by miR-216a demethylation (N = 3, *P < 0.05). **(B)** Up-regulating HMGB3 counteracts the effects of miR-216a demethylation on promoting Caspase9, Caspase 3, E-cadherin, Bax, and inhibiting Bcl2 and N-cadherin, compared with 5’-AZ group (N = 3, *P < 0.05). **(C)** Up-regulating HMGB3 counteracts the inhibition of miR-216a demethylation on invasion and migration (N = 3, *P < 0.05). **(D)** Up-regulating HMGB3 counteracts the inhibition of miR-216a demethylation on cell viability, compared with 5’-AZ miR-216a group (N = 3, *P < 0.05). **(E)** Up-regulating HMGB3 counteracts the inhibition of miR-216a demethylation on Wnt/β-catenin pathway, compared with 5’-AZ miR-216a group (N = 3, *P < 0.05). **p < 0.01.

Demethylation was carried out in TE-1 cell line with 5’-AZ in miR-216a group, and transfection of HMGB3 over-expression vector was additionally performed in 5’-AZ miR-216a+HMGB3 group. There was no difference in miR216a expression between the two groups; however, the 5’-AZ miR-216a+HMGB3 group showed higher HMGB3, decreased apoptosis, enhanced proliferation, as well as down-regulated Caspase 3, Caspase 9, E-cadherin, Bax, and up-regulated Bcl2, N-cadherin, Wnt/β-catenin pathway proteins (**Figure 5**). Therefore, up-regulation of HMGB3 eliminated the inhibition of miR-216a demethylation on HMGB3 and promoted cancer cell proliferation, invasion, and migration.

## Discussion

miR-216a has been implicated in the processes of cellular senescence and cardiovascular diseases (12). Moreover, it was identified as a negative regulator of breast cancer by modulating stemness properties and tumor microenvironment (24). It could suppress the proliferation and migration of human breast cancer cells *via* the Wnt/β-catenin signaling pathway. The regulatory effect of miR-216a has also been identified in esophageal squamous cell carcinoma by targeting TCTN1 to inhibit cell proliferation and induce apoptosis (15). In this study, we found that miR-216a binds to the 3’UTR of HMGB3 mRNA and downregulates its expression. In EC tissues and cell lines, the promoter region of miR-216a was hypermethylated, resulting in decreased expression of miR-216a and up-regulation of HMGB3, and thus promoted the proliferation and invasion of EC cells. Su et al. (24) stated that Limonin was a new approach to relieve hypermethylation of miR-216a and inhibited the growth of breast cancer. Our study highlighted the importance of DNA methylation of miR-216a in the progress of tumors, and indicated that miR-216a demethylation is valuable for targeted therapy of EC.

High mobility group-box 3 (HMGB3) is a recently identified member of high mobility group box subfamily. The well-studied members in this subfamily, including HMGB1 and HMGB2, exhibited important functions in various cancer progression (25). HMGB3 shares high protein sequence similarity with HMGB1 and has been shown to regulate hematopoiesis by fine tuning the balance of proliferation and differentiation and interacting with Wnt signaling pathway (16). Besides, overexpression of HMGB3 has been linked to acute myeloblastic leukemia (26) and metastatic breast cancers (22). Patients with increased HMGB3 expression were found to have poor survival (22). Our data are consistent with previous studies in some other cancer types, including breast cancer and lung cancer.

The activity of Wnt/β-catenin proteins was found to be significantly reduced after down-regulating HMGB3, suggesting that miR-216a regulates EC cells *via* Wnt/β-catenin pathway. Zhang et al. pointed out that HMGB3 positively regulated the activity of Wnt/β-catenin pathway by acting as its upstream protein (27). Wnt/β-catenin pathway is an important tumorigenesis pathway regulating biological processes in cells (28–30). When HMGB3 was down-regulated by miR-216a demethylation, Wnt/β-catenin pathway located downstream of HMGB3 lost its activity correspondingly. And decreased Wnt/β-catenin pathway activity directly led to malignant proliferation and metastasis of EC cells.

In this study, the relationship between miR-216a methylation and HMGB3 in EC cells was identified. Hypermethylation of miR-216a promoted up-regulation of HMGB3, while up-regulation of HMGB3 induced malignant proliferation and metastasis of cells. In the following study, the miR-216a/HMGB3 axis will be explored in depth, and we will explore whether the regulation of miR-216a demethylation on HMGB3 can improve the drug efficacy, and whether miR-216a/HMGB3 axis plays a role on early diagnosis and prognosis of patients with EC.

## Conclusion

In conclusion, up-regulation of miR-216a is found to inhibit malignant proliferation and metastasis of EC cells and induce apoptosis by down-regulating HMGB3. Our study provided novel drug target for the inhibition of EC progression and metastasis.

## Data Availability Statement

The original contributions presented in the study are included in the article/**Supplementary Material**. Further inquiries can be directed to the corresponding author.

## Ethics Statement

The studies involving human participants were reviewed and approved by Qilu Hospital of Shandong University. The patients/participants provided their written informed consent to participate in this study.

## Author Contributions

C-XS contributed to the conception or design of the work. FZ contributed to the acquisition, analysis, or interpretation of data for the work. LQ drafted the manuscript and critically revised the manuscript. All gave final approval and agree to be accountable for all aspects of work ensuring integrity and accuracy. All authors contributed to the article and approved the submitted version.

## Funding

The project was supported by the Natural Science Foundation of Shandong Province (ZR2020QH287).

## Conflict of Interest

The authors declare that the research was conducted in the absence of any commercial or financial relationships that could be construed as a potential conflict of interest.
