# Supplementary material for: Demethylated miR-216a Regulates High Mobility Group Box 3 Promoting Growth of Esophageal Cancer Cells Through Wnt/β-Catenin Pathway
Source: Front Oncol. 2021 Mar 23;11:622073. doi: 10.3389/fonc.2021.622073 (PMC8025835; doi:10.3389/fonc.2021.622073)
Supplement: Supplementary file 1 [file DataSheet_1.docx]

**Supplementary figures**


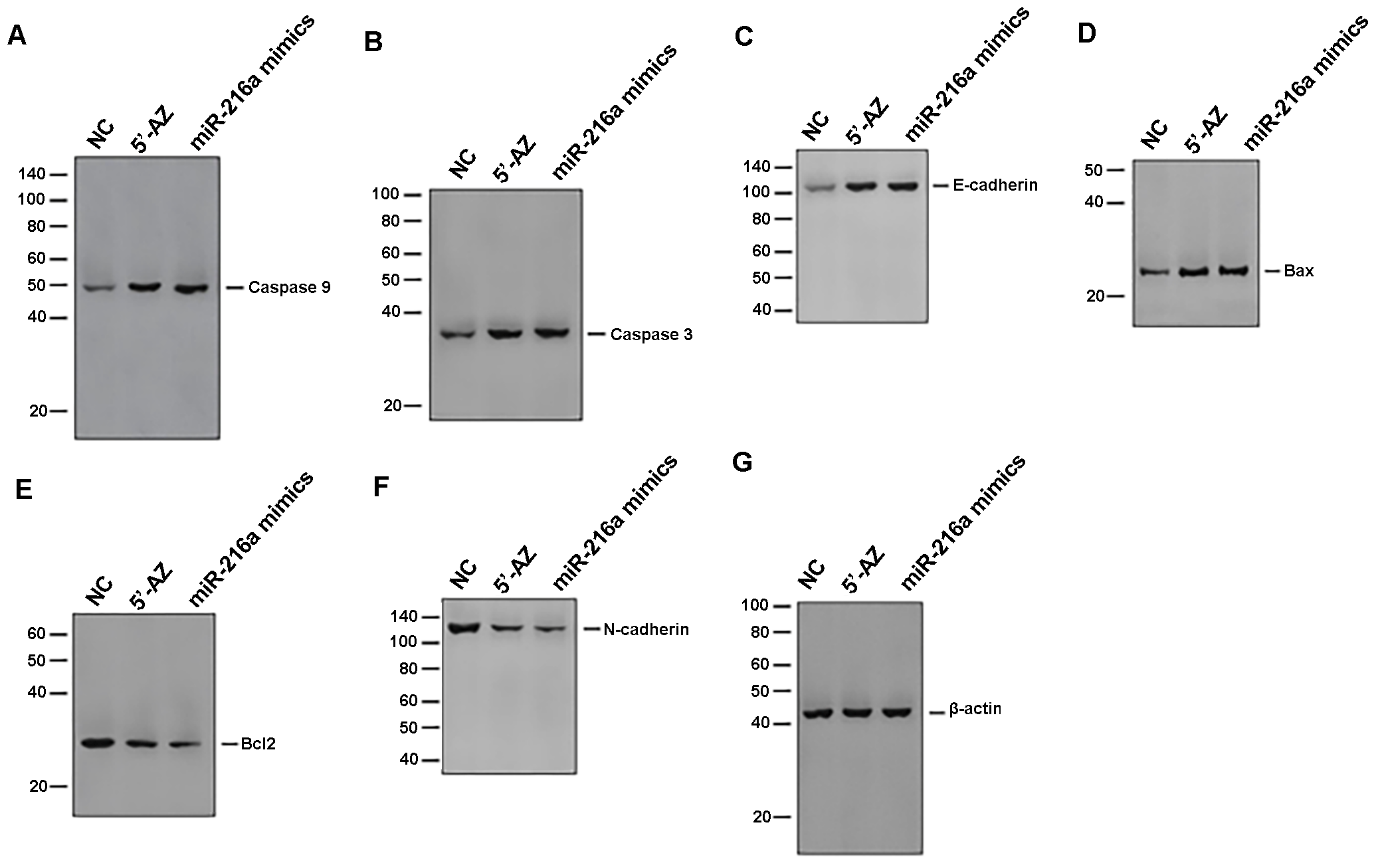


**Sfigure 1. The unprocessed western blot data of figure 3 panel C.**


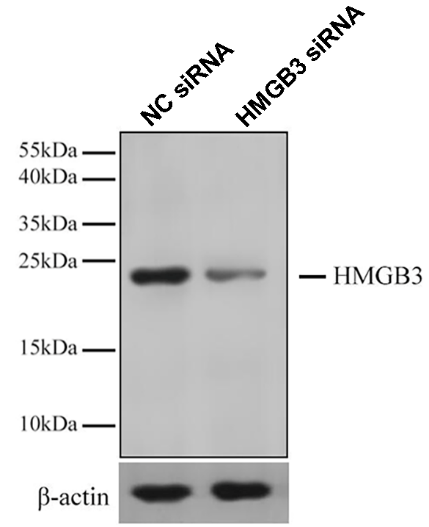


**Sfigure 2. The unprocessed western blot data of figure 4 panel A.**


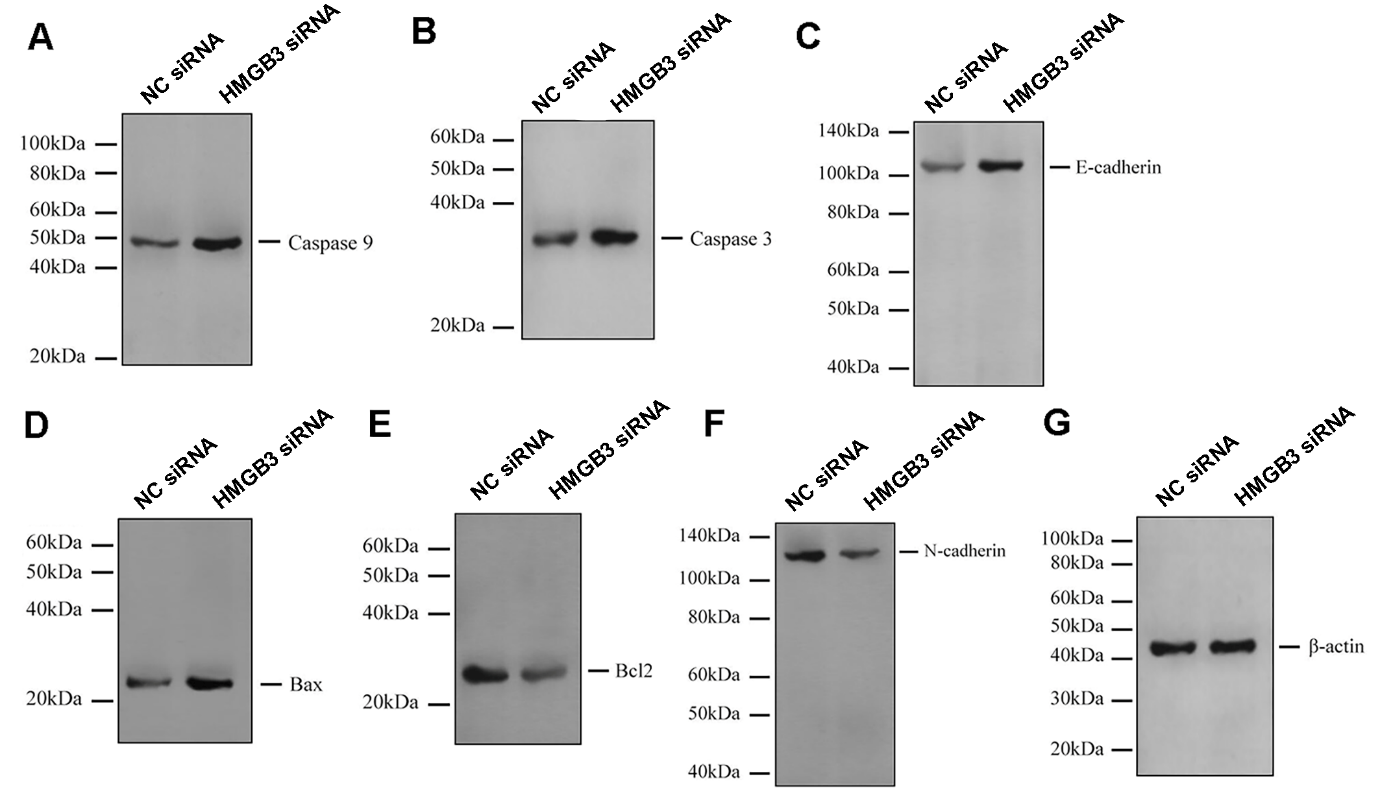


**Sfigure 3. The unprocessed western blot data of figure 4 panel B.**


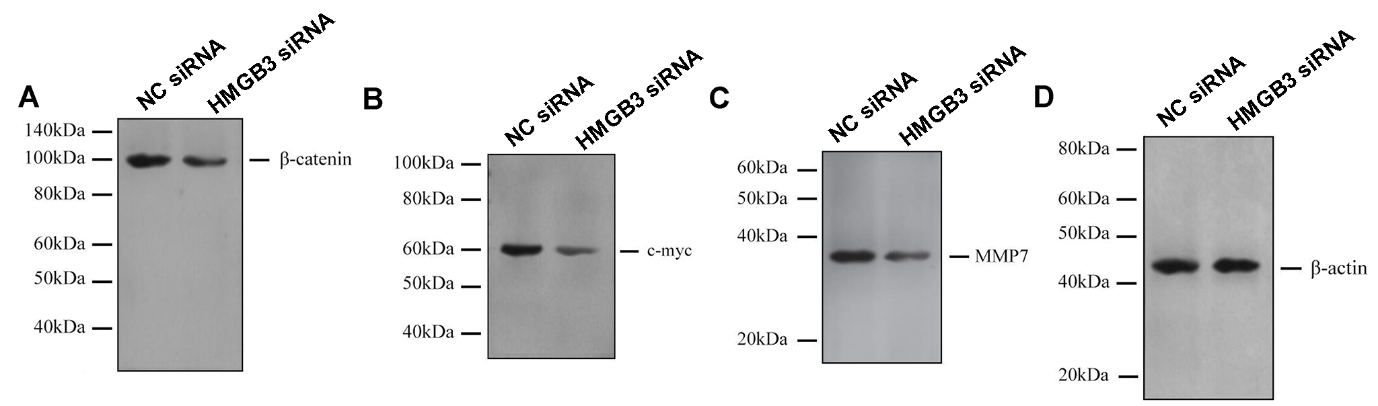


**Sfigure 4. The unprocessed western blot data of figure 4 panel D.**
